# The Impact of Attachment on Depression in Married Seniors: Chain Mediating Effects of Self-Esteem and Rumination

**DOI:** 10.3389/fpsyg.2021.677015

**Published:** 2021-08-12

**Authors:** Jiaxi Peng, Jiaxi Zhang, Kangwei Zhao, Xin Wang, Yi Wu, Peng Fang, Jing Lv

**Affiliations:** ^1^College of Teachers, Chengdu University, Chengdu, China; ^2^Department of Political Theory, Xi'an Research Institute of High Technology, Xi'an, China; ^3^Department of Military Medical Psychology, Air Force Medical University, Xi'an, China; ^4^The Second Medical Center & National Clinical Research Center for Geriatric Diseases, Chinese PLA General Hospital, Beijing, China

**Keywords:** late-life depression, attachment anxiety, attachment avoidance, self-esteem, rumination, chain mediating effects

## Abstract

In this study, we discuss the effects of attachment on depression and the mediating roles of self-esteem and rumination in Chinese seniors. We assessed 431 using the Experiences in Close Relationships Inventory, the Rosenberg Self-Esteem Scale, the Ruminative Responses Scale, and the Short Form of Center for Epidemiologic Studies Depression Scale. Both attachment anxiety and attachment avoidance significantly predicted depression in seniors. Attachment anxiety and attachment avoidance positively predicted rumination but negatively predicted self-esteem. Structural equation models showed that rumination and self-esteem fully mediated the effects of attachment anxiety and attachment avoidance on depression. The attachment of seniors is related to depression, and self-esteem and rumination have chain mediating effects between attachment and depression.

## Introduction

The Chinese population is rapidly aging. By the end of 2019, there were 254 million people aged 60 years and above (18.1% of the total population) and 176 million people aged 65 years and above (12.6% of the total population). Although China reportedly has the largest number of seniors, most other countries with a large number of seniors are developed nations. China, in contrast, remains at a rudimentary stage of development (Wang et al., 2020). The older adult population in China suffers from multiple pressures, including a decrease in income, the degeneration of social functions, and weakened physical capabilities (Hildebrandt, 2018). Thus, Chinese psychologists are concerned about psychological problems in seniors, such as anxiety, loneliness, and depression (Li et al., 2018). Depression is one of the most common mental problems among older Chinese adults, and more than one-third of seniors in urban regions suffer from some degree of depression (Lijun, 2014; Li et al., 2019). Owing to the degradation of social support and physical functioning, seniors wish to be geographically closer to and have more support from their families, including children and partners. This study aimed to evaluate the effects of attachment on depression and the mediating roles of self-esteem and rumination.

### The Effect of Attachment on Depression

Research concludes that depression is correlated with attribution style (Abdollahi et al., 2018), diseases (Hörnsten et al., 2016), negative life events (Assari and Lankarani, 2016), social support (Wang et al., 2014), personal characteristics (Zanarini et al., 2019), and cognitive-emotional regulation (Sloan et al., 2017). The attachment style is a key factor that impacts depression (Simon et al., 2019). Initially, attachment referred to the emotional connection between an infant and caregivers and the internal working model or cognitive schema that developed from this interaction. Such a model continues throughout life, persistently affecting the physical and mental health of individuals (David and Bearden, 2017; Del Giudice, 2019; Peng et al., 2019b). Ongoing research on attachment has demonstrated that subjects were not limited to the major caregivers at an early stage of life, but that other people (e.g., family members, loving partners, teachers, and friends) can all become subjects of attachment (Fraley and Roisman, 2019). Attachment is regarded as the individual development from “the cradle to the tomb” (Martins and Gaffan, 2010).

Like infants and young children, adults tend to find and develop intimate relationships and maintain contact and communication with others (Sommer et al., 2017). Adult attachment includes attachment avoidance and attachment anxiety (Cameron et al., 2012). Attachment avoidance refers to inadaptation caused by intimacy with a subject, and attachment anxiety is worry about being separated from or abandoned by the attached subject (Peng et al., 2020b). Both dimensions can be divided into high and low levels: high levels of attachment anxiety and attachment avoidance are considered insecure attachment and low levels of attachment anxiety and attachment avoidance are called secure attachment (Baer and Martinez, 2006). Chinese traditional culture is rooted in the concept of raising children to care for their parents in old age, which makes natural attachment with their children an inherent ideology for seniors in China (Wei et al., 2011, 2020). However, collectivism objectively requires Chinese seniors to establish intimate social links with other relations (Chui and Leung, 2016). Thus, the attachment of Chinese seniors is very important to their mental health, which has unique cultural roots.

Many studies imply that insecure attachment is significantly associated with depression (McMahon et al., 2005; Peng et al., 2013, 2020a; Scheffold et al., 2018). Any insecure attachment was significantly related to clinical depression in adults (Bifulco et al., 2002). Secure attachment significantly alleviates anxiety and depression in teenagers (Muris et al., 2001). In patients with initial symptoms of depression, insecure attachment can significantly predict the recurrence of depression after seven years (Conradi et al., 2018). Although abundant research confirms the relationship between attachment and depression, studies on the effects of attachment on depression among seniors are lacking. Molinari et al. (2001) studied attachment and interpersonal adaptation among people over age 65 and found that the attachment mode at the senile stage was significantly correlated with the attachment at the adolescence and early adult stages. Those with secure attachment have high self-esteem, self-efficacy, willingness to accept others, less loneliness, and a greater social support network and can successfully eliminate stress from life events. Hence, they demonstrate significantly higher levels of well-being and life satisfaction compared to those who exhibit insecure attachment (Molinari et al., 2001). Owing to the continuity of attachment development, attachment and depression are significantly correlated among seniors, as in other age groups.

The old-age stage is associated with a decline in physical functions and psychological resources. Seniors also depend more on their intimates (e.g., children and partners), indicating the importance of their attachments (Antonucci et al., 2004). According to the socio-emotional selectivity theory, seniors may perceive that their life years are is limited as they become older, and therefore, they are more likely to seek deep emotional meaning in the present, rather than make new friends (Carstensen et al., 2003; Löckenhoff and Carstensen, 2004; Kirkegaard Thomsen et al., 2017). In Asian cultures, people have high regard for families and intimate relations, and attachment plays a significant role in the mental health of older Chinese adults. However, with the development of China's social security system, Chinese seniors' economic and social life is becoming more independent (Wong et al., 2006). Some may find it unnecessary to maintain close relationships with others. Familial attachments have a significant effect on a senior's physical and psychological health. We propose that attachment is related to depression in seniors (hypothesis one).

### The Mediating Effect of Self-Esteem Between Attachment and Depression

The relationship between attachment style and depression and the underlying mental mechanism should be explored. The theory of attachment maintains that interactions with intimate relations can portray an individual's ego, including whether they believe they are worth being loved. If an individual can consistently receive care and help from others, they are likely to believe that they are worthy and that other people are dependable. If they cannot, they are likely to believe that they are worthless and that others are unreliable (Zeifman and Vassar, 2019). Insecure attachments will lead to the feeling of low capability and low value, which will result in low self-esteem (Barnum and Perrone-McGovern, 2017). Those with secure attachments have higher self-esteem compared to those with insecure attachments (Lee and Hankin, 2009). Wu further proved that attachment anxiety and attachment avoidance can directly affect self-esteem (Wu, 2009). Zhang et al. found that the insecure attachment of seniors was related to low self-esteem, and that self-esteem can, in part, mediate the effect of attachment on one's well-being (Zhang et al., 2016).

Low self-esteem is considered an important factor that can impact depression (Bajaj and Pande, 2016). In the Beck Depression Inventory, the susceptibility of individuals to depression was added to the ego and negative concepts of other people (Hemert et al., 2002). Patients with depression typically manifest with self-humiliation, self-accusation, self-crime, lower self-efficacy, and low self-esteem (Smeijers et al., 2017). Orth validated the six theoretical models of the relationship between low self-esteem and depression. The results supported the vulnerability model, which stated that (1) self-esteem is a very significant personality trait related to depression, (2) low self-esteem is an important risk factor for depression, and (3) strengthening self-esteem can ameliorate the symptoms of depression (Orth and Robins, 2013).

Moreover, according to the self-esteem theory of depression, low self-esteem is an important susceptible factor of depression, and negative life events can erode self-esteem and destroy an individuals' psychological protection system, thereby leading to depression (Hankin et al., 2007). An empirical study indicated that self-esteem correlated significantly and negatively with depression in seniors (Raj, 2018). Kwon and Ko (2017) demonstrated that self-esteem correlated significantly and negatively with depression among in-hospital patients with senility and can partially mediate its impact on depression. According to the literature, the insecure attachment style resulted in negative self-evaluation (namely, low self-esteem), which may be one cause for depression. Hence, we present that self-esteem can mediate the effect of attachment style on depression in seniors (hypothesis two).

### The Mediating Effect of Rumination Between Attachment and Depression

Insecure attachments function as a predictor for low self-esteem, whereas low self-esteem may result in the development of depressive symptoms. However, rumination may confound the relationships between attachment and depression and there may be other mediating processes between attachment and depression (Klein et al., 2011).

Rumination, initially proposed by Nolen-Hoeksema in the response styles theory (Nolen-Hoeksema, 1991), means that an individual unconsciously and repeatedly ponders over the cause, effect, consequence, and experienced emotions of negative life events but rarely thinks positively of solutions, which constitutes a negative thinking mode (Nolen-Hoeksema and Lyubomirsky, 2008; Wisco and Nolen-Hoeksema, 2010). Many cross-sectional, longitudinal, and experimental studies have proven that rumination functions as a risk factor for the development of depressive symptoms (McLaughlin et al., 2007; Carnevali et al., 2018). Philippot and Agrigoroaei found that repetitive thinking can significantly predict depression in seniors (Philippot and Agrigoroaei, 2017). Research has also found out that nervousness correlated positively with anxiety and depression among seniors and that rumination can mediate the effects of nervousness on anxiety and depression in seniors (Chen et al., 2020).

Many studies have verified the effects of attachment on rumination. For instance, insecure attachment styles correlated significantly and positively with rumination, and rumination mediated the relationship between insecure attachments and depression (Burnette et al., 2009). Insecure attachment styles were significantly and positively correlated with rumination and self-criticism among college students (Flett et al., 2020). There is currently no study on the relationship between attachment and rumination among seniors. We present that rumination can mediate the effects of attachment style on depression in seniors in a chain mode (hypothesis three).

**Figure 1:**
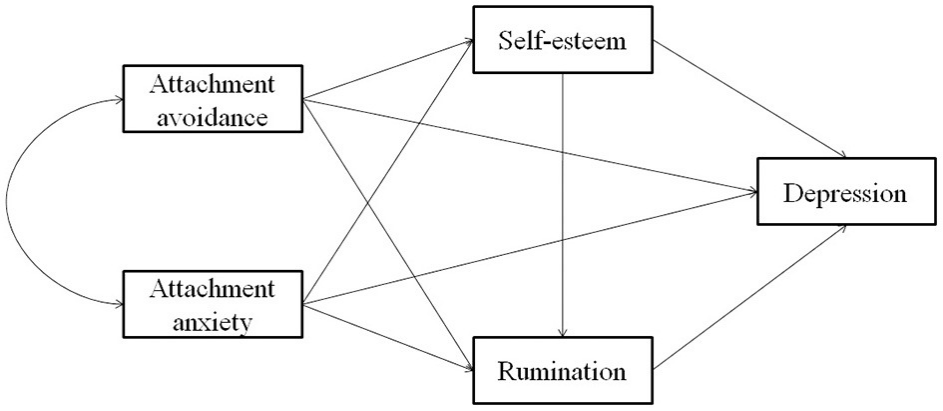
Hypothesis model of the current study.

Although the pairwise relationships between attachment, self-esteem, or rumination and depression have been investigated, there has been no study on the relationship of these four variables within the same model. In particular, considering the close relationship between self-esteem and rumination, those with low explicit self-esteem tend to focus more on their defects and shortcomings and more sufficiently encode negative information, forming more negative emotions manifested with more rumination (Tafarodi et al., 2003). A longitudinal study has shown that rumination can mediate the effect of self-esteem on depression (Kuster et al., 2012), self-esteem and rumination may have a chain mediation effect between attachment style and depression. The following hypothetical model (Figure 1) was presented in this study.

## Methods

### Participants

Through convenient sampling, a total of 431 seniors from eight large communities in Chengdu city, China were enrolled in our study. Given the probable relationship between attachment and marriage, we only included older married people who were not divorced or widowed, and only one senior was chosen from each family. Four hundred and one questionnaires were distributed, and 421 valid copies were returned (10 seniors failed to complete all answers). Altogether, there were 215 males and 216 females included in our analysis. The education level included elementary and below (*N* = 98), junior high (152), senior high (148), and junior college and above (33). The participants of the valid copies ranged in age from 60–70 years old, with a mean age of 64.48 (*SD* = 2.13) years. All participants were retired and had a regular income.

The testers involved 18 postgraduate or undergraduate students majoring in psychology, and they collected the data individually. The testers received intensive training before data collection. When a participant could not understand an item, the corresponding tester would offer a detailed explanation. For those participants with low education levels, the testers would read and explain each item and then select appropriate items according to the answers provided. Participants received a carton of eggs as compensation.

This study was approved by the Committee on Human Experimentation at the Chengdu University.

### Instruments

#### Experiences in Close Relationships Inventory (ECR)

This highly reliable and valid scale was compiled by Brennan and colleagues and translated into Chinese by Li and Kato (Tonggui and Kazuo, 2006). ECR consists of two subscales (attachment anxiety and attachment avoidance) involving 18 items for each subscale, such as “I worry about being abandoned” and “I am nervous when partners get too close to me.” Item responses ranged from 1 = strongly disagree to 7 = strongly agree. Of the 36 items, nine were reverse keyed. The mean score of each subscale was expressed as the score of the corresponding dimension, and a larger score indicated a higher level of attachment anxiety or attachment avoidance. As shown in Table 1, the Cronbach's alpha coefficient of ECR in the current study was 0.81 and 0.86 for attachment avoidance and attachment anxiety, respectively.

**Table 1:** Descriptive statistics and correlation analysis of all the variable (*n* = 421).

	**Mean**	**SD**	**1**	**2**	**3**	**4**	**5**
1. Attachment avoidance	3.04	0.61	(0.81)				
2. Attachment anxiety	3.52	0.65	0.25^**^	(0.86)			
3. Self-esteem	3.04	0.44	−0.23^**^	−0.35^**^	(0.86)		
4. Rumination	1.99	0.47	0.25^**^	0.37^**^	−0.39^**^	(0.80)	
5. Depression	1.93	0.68	0.24^**^	0.32^**^	−0.51^**^	0.54^**^	(0.92)

** *p < 0.01; The values in bracket are Cronbach's alpha coefficients*.

#### The Rosenberg Self-Esteem Scale (RSES)

The scale compiled by Rosenberg involves 10 items (five items were scored reversely) with single dimensions. The items included “On the whole, I am satisfied with myself” and “All in all, I am inclined to feel that I am a failure.” Item responses ranged from 1 6 =strongly disagree to 4 6 =strongly agree. The average score of the items was the score of this scale, with a larger score indicating a higher level of self-esteem (Robins et al., 2016). RSES was translated into Chinese with proven high reliability and validity (Zhang et al., 2019). As shown in Table 1, the Cronbach's alpha coefficient of RSES in this study was 0.86.

#### Ruminative Responses Scale (RRS)

RRS proposed by Nolen-Hoeksema consists of 22 items, including “Think about how sad I feel” and “Analyze recent events to try to understand why I am depressed” (Nolen-Hoeksema, 2000). Each item is rated on a scale ranging from 1 (rarely) to 4 (almost always). Han and Yang introduced RRS to various populations in China, with proven high reliability and validity (Xiu, 2009). The structure of RRS is under dispute. For instance, according to Bagby and Parker, RRS has two dimensions: symptom-focused rumination and self-focused rumination (Bagby and Parker, 2001). However, according to Treynor et al., RRS contains three dimensions: depressed-symptom rumination, reflective pondering, and brooding (Treynor et al., 2003). Notably, the total RRS score used in the current study, which is highly comparable to that of the original RRS (Raes et al., 2005), is the same approach employed in assessing seniors (Ekkers et al., 2011) but is not considered in calculating the three subscales as described by Treynor et al. (2003). Based on a previous study involving seniors, we used the mean value of all items as the RRS score (Chen et al., 2020). As shown in Table 1, the Cronbach's alpha coefficient of RRS in this study was 0.80.

#### The Short Form of Center for Epidemiologic Studies Depression Scale (10-item CES-D)

This scale, which was modified by Andresen et al. from the original CES-D, involves 10 items with a single dimension and has been widely used to evaluate depression among seniors (Andresen et al., 1994). The items include “I feel lonely” and “I was bothered by things that usually don't bother me.” Each item is rated on a scale ranging from 1 (rarely or never) to 4 (most or all of the time). Cheng and Chan found that the 10-item CES-D was highly effective among older Chinese adults (Cheng and Chan, 2010). As shown in Table 1, the Cronbach's alpha coefficient of the 10-item CES-D in the present study was 0.86.

#### Data Analysis

First, we analyzed the correlations among attachment anxiety, attachment avoidance, self-esteem, rumination, and depression. We conducted a regression analysis using depression as the dependent variable and attachment anxiety and attachment avoidance as the independent variables. We observed whether the regression coefficient of attachment over depression significantly changed after self-esteem and rumination were introduced, which was used as a criterion to evaluate whether self-esteem and rumination can impact the mediating effect of attachment on depression. Finally, we performed a structural equation model analysis with attachment anxiety and attachment avoidance as the independent variables, self-esteem and rumination as the mediator variables, and depression as the dependent variable.

All of the scales involved many items and we intended to control the inflated measurement errors due to multiple latent variables and improve measurement reliability and normality (Nasser-Abu Alhija and Wisenbaker, 2006). As such, we adopted the factorial algorithm method put forward by Rogers and Schmitt (2004) to create item parcels for all of the latent variables. We conducted the factor analyses of different variables and sorted the items according to the factor loads. Items ranking 1 and 2 were put into parcels A and B, respectively, and the items ranking 3 and 4 were put into parcels B and A, respectively. This method was repeated until all of the items were placed into appropriate parcels. The factor loads and variances of items within each item parcel were balanced, which reduced the differences between item parcels. The average value of each item parcel was then used as the observed variable to fit the latent variables of all study variables. We initially conducted a confirmatory factor analysis according to the two-step procedure proposed by Anderson and Gerbing (1988) to assess whether the measurement model conformed to the sampled data. We performed a structural model analysis.

## Results

First, the Harman single-factor test was used to determine whether there was a common method bias. All of the items in all of the scales were involved in the exploratory factor analysis, which demonstrated that 17 factors had eigenvalues larger than 1 and that the variance variation explained by the first factor was 18.74%, which was below the threshold of 40%. This indicated there was no evident common method bias.

We then performed a Pearson correlation analysis involving attachment anxiety, attachment avoidance, self-esteem, rumination, and depression (Table 1). Significant correlations were found between any two variables.

Then we conducted a regression analysis (Table 2). First, the results of the effect of participants' sociodemographic information on depression were calculated using the regression model, which showed that age, sex, and education have an insignificant impact on depression. Then, the influence of these sociodemographic variables was treated as a constant in the regression models. The relevant variables were subjected to regression analysis. In Model 1, self-esteem was the dependent variable, and attachment avoidance and attachment anxiety the independent variables. According to the regression analysis results, both attachment avoidance (β = –0.15, *p* < 0.01) and attachment anxiety (β = –0.32, *p* < 0.01) can significantly predict self-esteem. Similarly, the results of Model 2 indicated that both attachment anxiety (β = 0.33, *p* < 0.01) and attachment avoidance (β = 0.17, *p* < 0.01) can significantly predict rumination.

**Table 2:** Regression analysis (*n* = 421).

**Model**	**Dependent**	**Predictors**	**Model summary**		**Coefficients**			
			***F***	***R^**2**^***	**B**	**SE**	**β**	***t***
Constant	Depression	Age	2.16	0.01	−0.01	0.01	−0.03	−0.76
		Sex			0.07	0.05	0.08	1.65
		Education			−0.04	0.02	−0.07	1.23
1	Self-esteem	Attachment avoidance	36.27^**^	0.14	−0.11	0.03	−0.15	−3.30^**^
		Attachment anxiety			−0.21	0.03	−0.32	−6.80^**^
2	Rumination	Attachment avoidance	39.98^**^	0.16	0.13	0.04	0.17	3.56^**^
		Attachment anxiety			0.23	0.03	0.33	7.06^**^
3	Depression	Attachment avoidance	31.88^**^	0.13	0.19	0.05	0.17	3.69^**^
		Attachment anxiety			0.29	0.05	0.28	5.94^**^
4	Depression	Attachment avoidance	56.78^**^	0.29	0.15	0.05	0.14	3.19^**^
		Attachment anxiety			0.12	0.05	0.11	2.50^*^
		Self-esteem			−0.67	0.07	−0.43	−9.63^**^
5	Depression	Attachment avoidance	65.46^**^	0.32	0.11	0.05	0.10	2.27^*^
		Attachment anxiety			0.13	0.05	0.13	2.84^**^
		Rumination			0.68	0.06	0.47	10.74^**^
6	Depression	Attachment avoidance	70.11^**^	0.40	0.07	0.05	0.06	1.53
		Attachment anxiety			0.05	0.04	0.05	1.23
		Self-esteem			−0.51	0.07	−0.32	−7.59^**^
		Rumination			0.55	0.06	0.38	8.87^**^

* *P < 0.05*;

** *P < 0.01*.

Depression was the dependent variable in Model 3 and attachment anxiety and attachment avoidance the independent variables. The results showed that both attachment anxiety (β = 0.29, *p* < 0.01) and attachment avoidance (β = 0.19, *p* < 0.01) significantly predict depression. In Model 4, self-esteem was added to the regression equation between attachment and depression. The results showed that attachment avoidance (β = 0.14, *p* < 0.01), attachment anxiety (β = 0.11, *p* < 0.05), and self-esteem (β = 0.14, *p* < 0.01) significantly predicted depression. Similarly, in Model 5, rumination was added to the regression equation between attachment and depression. Attachment avoidance (β = 0.11, *p* < 0.05), attachment anxiety (β = 0.13, *p* < 0.01), and rumination (β = 0.68, *p* < 0.01) significantly predicted depression. However, after both self-esteem and rumination were introduced into the regression equation model, neither attachment anxiety (β = 0.05, *p* = 0.22) nor attachment avoidance (β = 0.06, *p* = 0.13) significantly predicted depression (Model 6).

**Figure 2:**
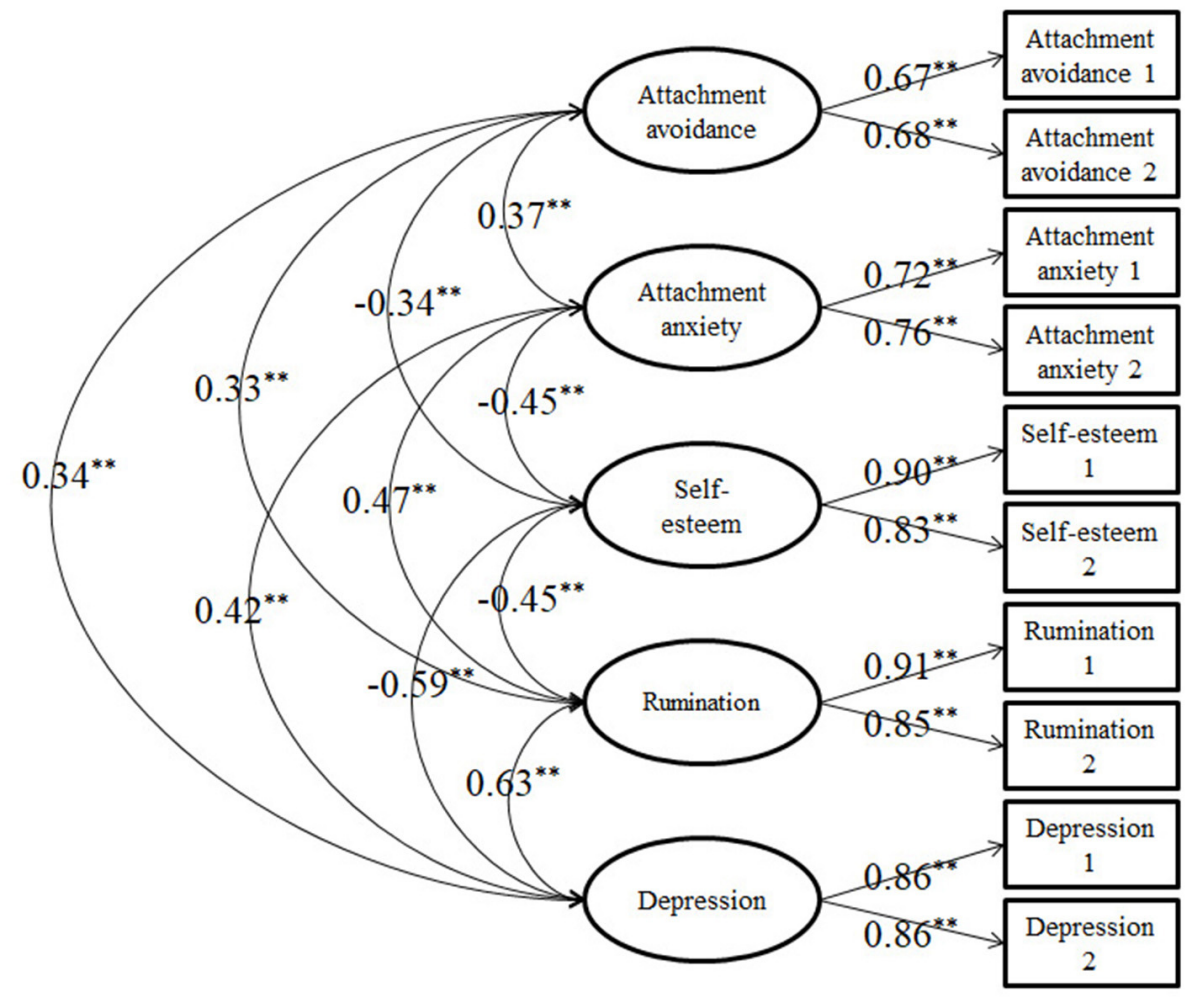
Measurement model. **P < 0.01.

We used structural modeling analyses to further explain the relationships between variables. First, we conducted a confirmatory factor analysis. The measurement model involved five latent variables (attachment anxiety, attachment avoidance, self-esteem, rumination, and depression) and 10 observational variables (10 created item parcels). The results demonstrated that the measurement model fits the data well: χ^2^*/df* = 1.09, RMSEA = 0.02, SRMR = 0.03, and CFI = 0.99. Each factor loading was significant, and all of the latent variables correlated significantly with each other (Figure 2).

We then performed a structural model analysis. The initial model was constructed based on the hypotheses. In the initial model, the independent variables were attachment anxiety and attachment avoidance, the mediator variables were self-esteem and rumination, and the dependent variable was depression. The results showed that both attachment anxiety and attachment avoidance affected self-esteem, rumination, and depression; self-esteem and rumination affected depression; and self-esteem affected rumination. The initial model was estimated and tested using the maximum likelihood method. The results proved that the model fits the sampled data well, but that the direct effects of attachment anxiety (β = 0.06, *p* = 0.30) and attachment avoidance (β = 0.03, *p* = 0.68) on depression were insignificant, suggesting that the effect of attachment on depression can be fully mediated by self-esteem and rumination.

**Figure 3:**
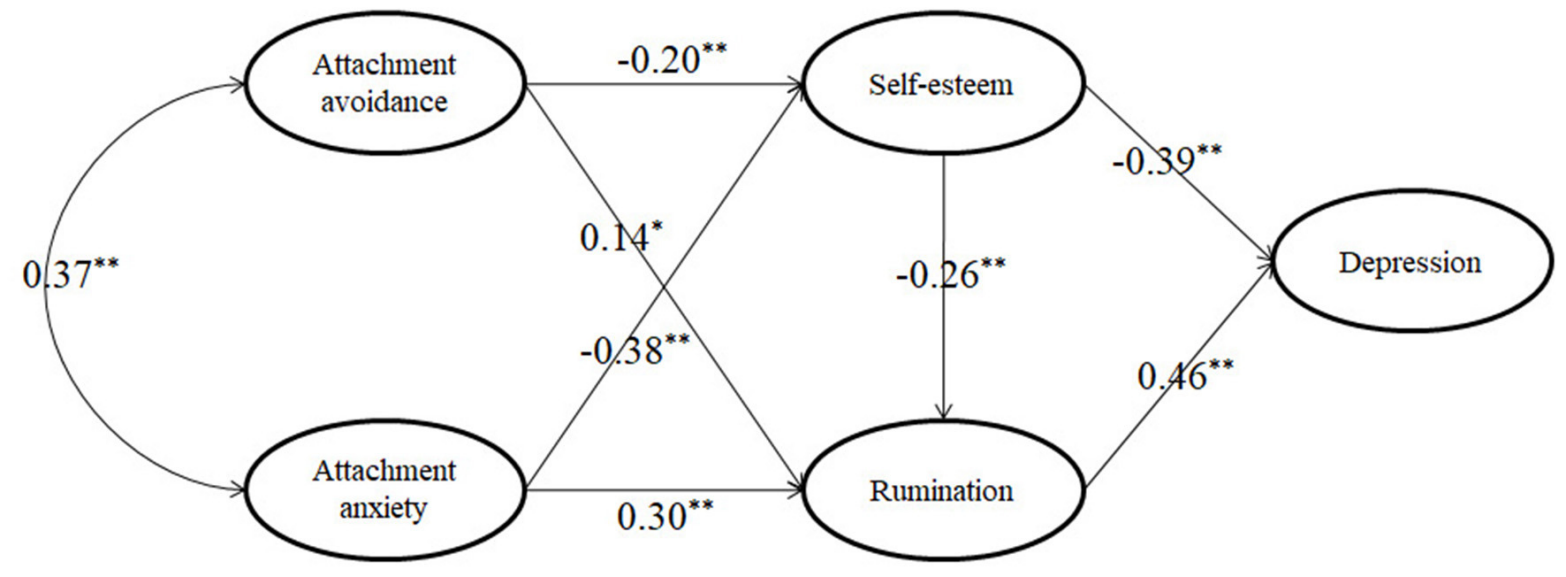
Structural model. *P < 0.05; **P < 0.01.

In the final model, we excluded the insignificant paths. The final model fit the sampled data well: χ^2^*/df* = 1.06, RMSEA = 0.01, SRMR = 0.02, and CFI = 0.99 (Figure 3). Finally, as shown in Table 3, we calculated the standardized direct, indirect, and total effects of the final model. The results demonstrated that the 95% confidence interval of all of the effects does not overlap with zero, which indicated that all direct and indirect effects were significant (*p* = 0.05). It also confirmed the mediating roles of self-esteem and rumination in the relationship between attachment and depression.

**Table 3:** Standardized effect and 95% CIs for the final model.

**Model pathways**	**Estimated effect**	**95% CI**	
		**Lower bonds**	**Up bonds**
**Direct effect**			
Attachment avoidance → Self-esteem^a^	−0.20	−0.35	−0.04
Attachment avoidance → Rumination^a^	0.14	0.02	0.28
Attachment anxiety → Self-esteem^a^	−0.38	−0.51	0.23
Attachment anxiety → Rumination^a^	0.30	0.14	0.48
Self-esteem → Rumination^a^	−0.26	−0.43	−0.11
Self-esteem → Depression^a^	−0.39	−0.54	−0.24
Rumination → Depression^a^	0.46	0.34	0.59
**Indirect effect**			
Attachment avoidance → Rumination^a^	0.05	0.01	0.11
Attachment avoidance → Depression^a^	0.16	0.06	0.27
Attachment anxiety → Rumination^a^	0.10	0.04	0.18
Attachment anxiety → Depression^a^	0.33	0.23	0.44
Self-esteem → Depression^a^	−0.12	−0.21	−0.05
**Total effect**			
Attachment avoidance → Self-esteem^a^	0.20	−0.35	−0.04
Attachment avoidance → Rumination^a^	0.19	0.03	0.34
Attachment avoidance → Depression^a^	0.16	0.06	0.27
Attachment anxiety → Self-esteem^a^	−0.38	−0.51	−0.23
Attachment anxiety → Rumination^a^	0.41	0.26	0.55
Attachment anxiety → Depression^a^	0.33	0.23	0.44
Self-esteem → Rumination^a^	−0.26	−0.42	−0.11
Self-esteem → Depression^a^	−0.51	−0.64	−0.37
Rumination → Depression^a^	0.46	0.34	0.59

a *Empirical 95% confidence interval does not overlap with zero*.

## Discussion

In this study, we adopted a chain mediating effects method to explore the impact of self-esteem and rumination on attachment's effects on depression in Chinese seniors. The results supported the hypotheses of the current study and confirmed the mediating roles of self-esteem and rumination in the relationship between attachment and depression.

Concordant with our hypothesis, one key finding was the positive correlation of depression with both attachment anxiety and attachment avoidance—a conclusion that has been documented in many studies (McMahon et al., 2005; Scheffold et al., 2018). We extended this conclusion to seniors. Attachment anxiety reflects an individual's worry that they may be rejected and abandoned by someone. Attachment avoidance means that the individual feels uncomfortable about intimate behaviors with others. Insecure adult attachment is manifested as high attachment anxiety and/or high attachment avoidance. Both the feeling of loss due to the fear of being rejected and the sense of alienation due to the fear of intimacy can result in depression (Baker and Verrocchio, 2016). Attachment is essentially an individual's perception and determination concerning whether he is worth having relationships with other people and whether he is valued by other people (Blackwell et al., 2017).

According to the socio-emotional selectivity theory, seniors who are in the late stage of the life cycle and with degenerate physiological and social functions place more value on the present attachment relationship. They fear not being cared for by others and emotional alienation (for example, from children or a spouse). The internal working models can encourage negative impressions about themselves and others, which leads to feelings of helplessness, hopelessness, unattractiveness, and rejection (Cicchetti and Toth, 1998). Such internal working models may affect the physiological functions of emotion modulation and emotion expression and may have a considerable impact on depression development (Cicchetti and Toth, 1998; Spangler and Zimmermann, 1999). Under the influence of cultural predilections for the community over individualism and the traditional Chinese concept of children caring for elderly parents, Chinese seniors must maintain a sense of psychological intimacy with others (Chen et al., 2017). According to socio-emotional selectivity theory, the deprivation of psychological needs is likely to bring emotional imbalances and impact psychological health. Both attachment dimensions are closely related to the depressive symptoms of Chinese seniors.

Another important finding was the chain mediation effect of self-esteem and rumination in the relationship between attachment style and depression in Chinese seniors. This is the major theoretical contribution of this study. This study first confirmed the hypothesis that self-esteem mediates the effect of attachment style on depression in Chinese seniors. The Internal Working Model of Attachment maintains that attachment, as the emotional connection between an individual and a subject, affects psychological and social adaptations through the internal working model (Roberson et al., 2011). This internal working model includes the individual's self-evaluation and perceptions according to the outside world (Shemmings, 2006). Individuals with a secure attachment, or those with low attachment anxiety and low attachment avoidance, do not repel or fear the loss of intimate relationships. Rather, they think they are attractive and trustworthy, so they have higher self-assessment and self-esteem. According to the self-esteem theory and the susceptibility model of depression, when an individual has high self-esteem, they have more psychological response resources and can better handle problems, which alleviates psychological injuries due to negative life events. This helps prevent depression (The role of self-esteem instability in the development of postnatal depression: A prospective study testing a diathesis-stress account, 2015).

This study also supports the hypothesis that attachment styles can affect depression through rumination mediation. Seniors with higher attachment anxiety and attachment avoidance will have lower self-esteem, higher rumination, and more severe depression. Seniors are typically eager to establish intimate relationships and maintain communication with others. If such demands cannot be satisfied, they will harbor a negative self-assessment. Low self-esteem will make them more sensitive to negative information, forming more negative memories. Consequently, they tend to focus and ruminate on their shortcomings, which causes a depression state that leads to a decline in physical capabilities, degradation of social functions, and aggravation of depression (Thomsen et al., 2004; Peng et al., 2019a,b; Lisa et al., 2020). Furthermore, the internal working model is a cognitive scheme or cognitive style. Rumination, a negative cognitive style, consists of repetitive and compulsive thinking, negative deduction and attribution, malfunctioned attitudes, hopelessness, pessimism, and self-criticism (Geoffrey, 2010). The cognitive model of depression maintains that depression originates from a negative cognitive style (Rief and Joormann, 2019), which is influenced by insecure attachment and rumination.

As China's population ages, our study results have practical implications to help lower seniors' risk of depression and promote their mental health. Seniors with insecure attachments should expand their interpersonal interactions and keep close contact with their friends and family, which will decrease their tendency to avoid other people, reduce their fear about the loss of intimate relationships, and reduce depressive emotions. Children in China should provide emotional support for their parents to help them establish secure attachments and protect again geriatric depression symptoms. However, per positive psychology advocates (Mitchell et al., 2010), seniors' self-assessment must be improved (Seligman et al., 2005) by participation in strength and physical exercises, construction of an atmosphere that supports elders, and rumination reduction.

Our study has several limitations. First, this cross-sectional study does not uncover the causality between variables; specifically, it is difficult to justify the indirect effects of attachment and depression via rumination or self-esteem using only data at a single time point. Future longitudinal research should be conducted to verify the mediating role of rumination and self-esteem in the relationship between attachment and depression. Second, attachment was regarded as a comprehensive and general emotional connection, but it should be further studied to explore whether other concrete types of attachment (husband*-*wife attachment or parent*-*child attachment) will similarly affect depression in seniors (Mallinckrodt and Wei, 2005). Future research should investigate the influence of marital or parent–child relationships through careful experimental design. Third, we enrolled only seniors in urban regions who were married and not divorced or widowed. Sociodemographic variables may not have been given enough consideration. Other relationships and sociodemographic variables may also be significantly related to depression. Additional studies are warranted to further understand the impact of sociodemographic variables and to evaluate the impact of senior depression.

## Data Availability Statement

The original contributions presented in the study are included in the article/supplementary material, further inquiries can be directed to the corresponding author/s.

## Ethics Statement

The studies involving human participants were reviewed and approved by the Committee on Human Experimentation at the Chengdu University. The patients/participants provided their written informed consent to participate in this study.

## Author Contributions

JP, PF, and JL conceived and designed the study. JP and JZ collected the data. JP, KZ, XW, and YW analyzed the data. JP and JZ contributed reagents, materials, and analysis tools. JP, PF, and JL wrote the manuscript. All authors contributed to the article and approved the submitted version.

## Conflict of Interest

The authors declare that the research was conducted in the absence of any commercial or financial relationships that could be construed as a potential conflict of interest.

## Publisher's Note

All claims expressed in this article are solely those of the authors and do not necessarily represent those of their affiliated organizations, or those of the publisher, the editors and the reviewers. Any product that may be evaluated in this article, or claim that may be made by its manufacturer, is not guaranteed or endorsed by the publisher.
